# Feeding styles and evening family meals among recent immigrants

**DOI:** 10.1186/1479-5868-10-84

**Published:** 2013-06-26

**Authors:** Alison Tovar, Erin Hennessy, Aviva Must, Sheryl O Hughes, David M Gute, Sarah Sliwa, Rebecca J Boulos, Emily Kuross Vikre, Christina Luongo Kamins, Kerline Tofuri, Alex Pirie, Christina D Economos

**Affiliations:** 1 Department of Nutrition and Food Science, University of Rhode Island, 112 Ranger Hall, Kingston, RI 02818, USA; 2 Health Behaviors Research Branch, Behavioral Research Program, Division of Cancer Control and Population Sciences, National Cancer Institute, 9609 Medical Center Drive, Bethesda, MD 20892, USA; 3 Dept. of Public Health and Community Medicine, Tufts University School of Medicine, 136 Harrison Ave, Boston, MA 02111, USA; 4 Children’s Nutrition Research Center, Baylor College of Medicine, 1100 Bates, Houston, TX 77030, USA; 5 Department of Civil and Environmental Engineering, School of Engineering, Tufts University, 113 Anderson Hall, 200 College Avenue, Medford, MA 02155, USA; 6 Friedman School of Nutrition Science and Policy at Tufts University, 150 Harrison Ave, Boston, MA 02111, USA; 7 Immigrant Service Providers Group/Health, 337 Somerville Ave, Somerville, MA 02143, USA

**Keywords:** Family meal, Feeding styles, Children, Obesity, Immigrants

## Abstract

The protective effect of family meals on unhealthy weight gain and diet has been shown across multiple age groups; however, it is unknown whether a similar effect is present among diverse immigrant populations. In addition, little research has focused on factors associated with the frequency of evening family meals, such as feeding styles (how parents interact with their child around feeding). Therefore the goals of this paper are to explore the 1) association between the frequency of evening family meals and child weight status among new immigrant families, and 2) influence of immigrant mothers’ feeding styles on the frequency of evening family meals.

Baseline self-reported socio-demographic information and measured heights and weights were collected for both mother and child (age range: 3–12 years) among 387 mother-child dyads enrolled in Live Well, a community-based, participatory-research, randomized controlled lifestyle intervention to prevent excessive weight gain in recent (<10 years in the U.S.) immigrant mothers and children. For children, height and weight measurements were transformed into BMI z-scores using age-and sex-specific CDC standards and categorized as overweight (85^th^–94^th^ percentile) and obese (≥95^th^ percentile); mothers’ BMI was calculated. Frequency of evening family meals, eating dinner in front of the TV, acculturation and responses to the Caregiver’s Feeding Styles Questionnaire (CFSQ) were also obtained from the mother. Children were categorized as “eating evening family meals regularly” if they had an evening family meal ≥5 times per week.

Overall, 20% of children were overweight and 25% were obese. Less than half (40.9%) of families had regular evening family meals. In multivariate analyses, adjusting for covariates, children who were overweight/obese were significantly less likely to have ≥5 evening family meals/week compared with normal weight children (OR = 0.51, 95% CI 0.32-0.82) . Mothers who had a low demanding/high responsive or a low demanding/low responsive feeding style, were less likely to have ≥5 evening family meals/week compared to mothers with a high demanding/high responsive feeding style (OR = 0.41, 95% CI 0.18-0.0.96, OR = 0.33, 95% CI 0.13-0.87, respectively). Future interventions and programs that seek to help parents establish healthy household routines, such as family meals, may consider tailoring to specific maternal feeding styles.

## 

Although there is evidence to suggest that rates of childhood obesity have stabilized among some populations, disparities among racial/ethnic populations still persist [1-3]. Immigrants, including immigrant children, become at greater risk for overweight and obesity with increased length of stay in the U.S. [4-10]. The challenges immigrant families face as they are exposed to a new culture are likely to have a direct impact on their health and wellbeing [10,11]. One challenge includes navigating the U.S.’s “obesogenic” environment. This environment is characterized by the high availability of inexpensive, energy-dense foods, and limited opportunities for either meal-oriented food preparation or sufficient physical activity, all of which have been shown to contribute to excessive weight gain [4-7,12-14]. Exploring the presence of protective routines, such as family meals, and the factors associated with them, such as responsive or more authoritative feeding styles, within a diverse cohort of new immigrants is important for the prevention of obesity in this population.

Family meals are associated with increased fruit and vegetable consumption, and better psychosocial health [15-18]. The protective effect of family meals has been shown across multiple age groups, including a nationally representative sample of preschool-age children, as well as among school-age children and adolescents [18-21]. However, it is unknown whether the effect of family meal frequency on weight and diet is similar among diverse immigrant populations. While little research has focused on factors associated with the frequency of family meals, especially among diverse populations, there are data to suggest the strong role of parental and familial dynamics, as they influence the occurrence and frequency of family meals [22]. Given the critical role parenting style and family dynamics play in determining children’s eating behaviors, habits, and attitudes, as well as the physical and social food environment of the home, it is not surprising that these factors influence the frequency of family meals [23,24]. For instance, a recent study found a positive association between maternal and paternal authoritative parenting style and frequency of family meals [25]. Exploring feeding style, as opposed to general parenting style, may be more helpful in predicting the frequency of family meals because of the context-specific impact of feeding [26]. The notion of feeding styles assumes that parents possess overarching styles that reflect how they interact with their children during feeding situations that can be described across two dimensions: demandingness and responsiveness. Demandingness refers to how much the parent encourages eating, whereas responsiveness refers to how parents encourage eating, that is, in a responsive or nonresponsive way. Based on the two dimensions, parents are categorized into four feeding style typologies: high demandingness/high responsiveness (i.e., actively encouraging eating using non-directive and supportive behaviors), high demandingness/low responsiveness (i.e., encouraging eating using highly directive, unsupportive behaviors), low demandingness/high responsiveness (i.e., making few demands on the child to eat and being warm and accepting) and low demandingness/low responsiveness (i.e., making few demands on the child to eat and being unsupportive) [27].

An increasing number of studies with low income, diverse families have found that children of parents who are responsive to their child’s emotional states but refrain from setting appropriate boundaries with their child (low demandingness/high responsiveness) are at the greatest risk for childhood obesity [27-32]. In addition, this same feeding style has been associated with poor diet quality [33,34]. However, the association between maternal feeding style and family meal frequency has not been explored; furthermore, it is unknown whether the beneficial effect of family meals on weight and diet is also present among diverse immigrant populations.

Given the unique situation that immigrant parents and children face as they acculturate to a new social, cultural and physical environment, the role of feeding styles may be particularly important in influencing the frequency of family meals. The goals of this paper are to test the following within a sample of recent immigrant families (living in the U.S. < 10 years): 1) the association between the frequency of evening family meals with child weight status and 2) the relation between maternal feeding styles and the frequency of evening family meals.

## Methods

### Study structure

The data analyzed for this study were collected at baseline from Live Well, a randomized controlled lifestyle intervention that utilizes a community-based participatory research (CBPR) approach. The central premise of Live Well is that an intervention co-created by community partners and academic researchers can prevent excessive weight gain in recent immigrants. Mother/child dyads were enrolled in the study and data from 387 dyads were collected at baseline. To be eligible for the study, the mother must have resided for <10 years in the U.S.; be of Haitian, Latino or Brazilian descent; be 20–55 years of age; not be pregnant (or ≥6 months postpartum); have a child between the ages of 3–12 years; live in the Greater Boston area; and be willing to be randomized. Informed consent was obtained for all participants: written consent from all caregivers as well as assent for children over 7 years of age. Mother-child dyads attended a measurement day at a nearby community center where they completed the assessments. The study protocol was approved by the Social, Behavioral and Educational Institutional Review Board of Tufts University.

### Measurements

#### Caregiver’s feeding styles questionnaire (CFSQ)

The CFSQ is a self-administered instrument measuring parenting approaches to child feeding (e.g., feeding styles) [27]. The feeding styles construct assumes that parents possess overarching styles that reflect how they interact with their children during feeding situations and are based on two dimensions. The demandingness dimension refers to *how much* the parent encourages eating, whereas the responsiveness dimension refers to *how* parents encourage eating, that is, in a responsive or nonresponsive way. Demandingness and responsiveness are derived through 7 child-centered and 12 parent-centered feeding directives measured on a 5-point Likert scale (ranging from never to always). *Child-centered feeding* promotes child autonomy (e.g., reasoning, complimenting, and helping the child to eat). *Parent-centered feeding* uses directives that control children’s eating through external pressure (e.g., demands, threats, and reward contingencies). Based on the two dimensions, parents are categorized into four feeding style typologies: high demandingness/high responsiveness (i.e., actively encouraging eating using non-directive and supportive behaviors), high demandingness/low responsiveness (i.e., encouraging eating using highly directive; unsupportive-behaviors), low demandingness/high responsiveness (i.e., making few demands on the child to eat and being warm and accepting) and low demandingness/low responsiveness (i.e., making few demands on the child to eat and being unsupportive) [27]. Information about the development, reliability, and validity of this instrument is published elsewhere [27,35] as well as information about the use of the CFSQ in this population [32].

In this study, feeding styles are described along the two dimensions of demandingness and responsiveness rather than the four more commonly used labels of authoritative, authoritarian, indulgent and uninvolved. The community partner members of the study’s steering committee expressed concern about the commonly used feeding style typology labels, and the potential for erroneous or judgmental ethnic-specific interpretations. The concern arose primarily from the labels themselves, as opposed to the inherent meaning of the typologies or the specific questions on the CFSQ. Therefore, the decision was made to use the two dimensions as descriptors, and avoid the application of the labels. This has been described previously [32].

### Evening family meals

Mothers were asked: “How many days per week does your family sit at a table to eat dinner together? (this includes eating with some or all of the family and occasions when it is just your child and you, this does not include eating dinner together in front of the television)” [36]. According to definitions used by others [15,18,37] and the response distribution for this question in our sample, we categorized children as having the routine of eating regular evening family meals if they had evening family meals ≥5 times per week.

### Demographics

Other variables of interest were collected through the self-administered (or interpreter-assisted) survey completed at baseline measurement. Mothers reported their child’s date of birth and gender, as well as their own age, race/ethnicity, marital status, education level, employment status, number of jobs worked, and household size and composition. Child’s country of birth, and the number of months the mother has lived in the U.S. were used as proxy measures for acculturation.

### Body mass index (BMI)

Height and weight were obtained from mothers and children. Measurements were taken in triplicate following standardized procedures [38]. Height was measured, without shoes, to the nearest eighth of an inch using a portable stadiometer (The Shorr Board vertical stadiometer, Shorr Production, Olney, MD). Weight was measured in light clothing, without shoes, to the nearest 0.5 lb on a portable digital scale (Befour PS-6600 Portable Scale; Befour Inc., Saukville, WI). Body mass index (BMI) was calculated from the average of the three body weight and height measurements for each dyad (kg/m^2^). Each child’s BMI was transformed into a z-score relative to the age- and sex-specific CDC reference [39]. The percentile score corresponding to the z-score, and the following terminology, were utilized to classify child weight categories: underweight (<5^th^ percentile), normal weight (≥5^th^ to <85^th^ percentile), overweight (≥85^th^ to <95^th^ percentile), and obese (≥95^th^ percentile) [40]. Maternal weight status was classified based on BMI using the categories: underweight (<18.5), normal weight (18.5–24.9), overweight (25.0–29.9), and obese (>30.0) [41].

### Statistical analysis

Descriptive statistics were used to characterize frequencies, including family meals overall and by socio-demographic characteristics (child’s age; maternal education, marital status, employment and number of jobs, and number of children in the household). We compared the prevalence of obesity in children exposed and not exposed to eating family meals ≥5 times per week. Statistical significance for these comparisons was based on a level of .05 using Chi-square tests.

According to the scoring procedure for the CFSQ [27], demandingness is calculated as the total mean score across all items (child-centered and parent-centered), while responsiveness is the ratio of child-centered items over the total score. Dichotomization at the median within the entire sample results in a typology of the different feeding styles. The median for demandingness in this population was 3.05 and for responsiveness was 1.11, which is similar to the median splits found among other samples [27,42-44].

For the multivariate models, logistic regression estimated the odds of child overweight and obesity associated with exposure to eating family meals ≥5 times/week. Findings are presented as unadjusted odds ratios (ORs) and ORs adjusted for ethnic group, child age, BMIz, and gender, and maternal education, employment status, marital status, BMI, and age. Multivariate logistic regression models also estimated the odds of eating as a family ≥5 times/week associated with the feeding style typologies. Based on previous research, a high responsiveness/high demandingness feeding style, often referred to in the literature as authoritative, was used as the referent category [28,29]. Interactions between length of time in the U.S. and feeding style, and ethnic group and feeding style were also tested. Final models adjusted for the following variables: ethnic group; child gender, age and BMIz; maternal age, education, employment, marital status, and BMI; these selections were based on previous research and whether a covariate was significantly associated with the outcome [15,18,37].

### Results

Overall, 20% of children were overweight and 25% were obese, resulting in a total of 45% who were at or above the 85th percentile. Fewer than half (40.9%) of families participated in evening family meals ≥5 times per week. In bivariate analyses, significant differences in child overweight and obesity were seen by maternal obesity and parental feeding style. Those mothers who were not obese were less likely to have an obese child (38.0% vs. 59.2%); those with a low demanding/high responsive feeding style had a higher percentage of overweight/obese children compared to other styles (55.8% vs. 40.7% for high demanding/high responsive, 39% for high demanding/low responsive and 44.1% for low demanding/low responsive) (Table 1).

**Table 1:** Prevalence of overweight and obesity and evening family meals according to socio-demographic characteristics

	**Overall population**	**Prevalence of overweight and obesity**	**Eating dinner as a family ≥5 times/week**
	**%**	**%**	**%**
**Child**			
Age (mean, sd)	6.2, 2.7	6.3, 2.7	5.9, 2.7
BMI-z (mean, sd)	0.9, 1.2	1.9, 0.7	0.8, 1.2
Gender			
% male	57.3	46.1	38.6
p value		0.18	0.19
**Mother**			
Ethnic group			
Brazilian	35.9	48.2	38.4
Haitian	34.6	40.3	35.9
Latino	29.5	47.4	50.0
p value		0.36	0.06
Education			
Less than high school	31.5	41.2	42.9
High school, trade/technical school	45.0	51.2	40.0
Some college/college graduate/graduate	23.5	40.5	39.3
p value		0.13	0.85
Employment status			
Employed full time >35 hrs/wk	24.0	48.3	33.7
Employed part time <35 hrs/wk	25.1	44.1	30.1
Employed seasonally/Unemployed	35.3	41.2	47.3
Student/Homemaker	15.6	53.5	53.5
p value		0.42	<0.01
Number of current jobs			
0	26.2	35.5	52.6
1	69.0	46.3	35.3
2	4.5	46.2	7.7
p value		0.27	<0.01
Marital status			
Never married	58.2	44.8	39.3
Married	21.3	41.3	37.5
Separated/Divorced/Widowed	20.5	50.7	45.5
p value		0.49	0.55
Number of children in the household <18			
1	34.2	46.0	39.5
2	43.0	50.0	39.7
≥3	22.9	39.8	39.7
p value		0.32	0.99
Length of time in U.S.			
<5 years	29.7	38.2	39.1
≥5 years	70.4	49.0	41.4
p value		0.06	0.68
Child’s place of birth			
U.S. born	43.2	45.6	42.9
Foreign born	56.7	46.2	38.1
p value		0.91	0.36
Maternal obesity (BMI cutoffs)			
<30	62.5	38.0	39.7
≥30	37.5	59.2	44.0
p value		<0.001	0.41
How often does your child eat dinner in a room with the TV turned on?			
A lot/sometimes	58.1	45.8	33.0
Rarely/never	41.9	45.8	53.6
p value		0.99	<0.0001
Feeding style			
High Demandingness/High responsiveness	15.6	40.7	52.5
High Demandingness/Low responsiveness	32.5	39.0	39.0
Low Demandingness/High responsiveness	34.0	55.8	38.8
Low Demandingness/Low responsiveness	17.9	44.1	36.8
p value		0.04	0.24

P-values generated from Chi-square tests and Fishers Exact for cells < 5.

Significant bivariate differences for families who ate family evening meals ≥5 times/week were seen by employment, number of jobs, and eating dinner in front of the TV. Differences by ethnicity were of marginal significance (p = 0.06). Latino families had more evening family meals ≥5 times/week compared to Brazilians and Haitians (50% vs. 38% and 36%, respectively). The Latina women in this study were from Central and South America (32% from El Salvador, 11% from Colombia, 9% from Guatemala, and 48% from other countries such as Dominican Republic, Bolivia, Peru and Honduras). Also, a higher percentage of mothers who were homemakers/students or who were unemployed had evening family meals ≥5 times/week (54% and 47%, respectively) compared with mothers who were employed full-time or part-time (33.7% and 30.1%, respectively). A higher percentage of those who did not have a job had evening family meals ≥5 times/week compared with those with one or two jobs (52.6% vs. 35.3% and 7.7%). Mothers who reported their child ate dinner in front of the TV rarely/never ate evening family meals ≥5 times/week more often than those that answered a lot/sometimes (53.6% vs. 33%).

In multivariate analyses, when adjusting for covariates, children who were overweight/obese were significantly less likely to have ≥5 evening family meals/week compared with normal weight children (OR = 0.51, 95% CI 0.32-0.82) (Table 2). We then explored the association between feeding dimensions and styles and frequency of evening family meals. Mothers who had either a low demandingness/high responsiveness or a low demandingness/low responsiveness feeding style were less likely to have ≥5 evening family meals/week compared with mothers who had a high demandingness/high responsiveness feeding style (OR = 0.41, 95% CI 0.18-0.0.96, OR = 0.33, 95% CI 0.13-0.87, respectively) (Table 2). We found no significant interaction between length of time in the U.S. and feeding style, and ethnic group and feeding style.

**Table 2:** Logistic regression analysis for family meals by overweight/obesity and feeding style

	**Family meals ≥5 times/week**				**Family meals ≥5 times/week**			
	**Unadjusted model**				**Adjusted model**			
Model 1: Overweight/Obesity*	OR	95% CI		p value	OR	95% CI		p value
Normal weight	referent				referent			
Overweight/Obese	0.61	0.40	0.9	0.02	0.51	0.32	0.82	<0.01
Model 2: Feeding style**								
High Demandingness/High Responsiveness	referent				referent			
High Demandingness/Low Responsiveness	0.58	0.31	1.10	0.09	0.49	0.21	1.09	0.08
Low Demandingness/High Responsiveness	0.60	0.31	1.07	0.08	0.41	0.18	0.96	0.04
Low Demandingness/Low Responsiveness	0.53	0.26	1.07	0.08	0.33	0.13	0.87	0.02

*Adjusted for ethnic group; child age and gender; maternal age, BMI, marital status, education, and employment status.

**Adjusted for ethnic group; child age, gender and BMIz; maternal age, BMI, marital status, and employment status.

### Discussion

The goals of this paper were to explore the association between the frequency of evening family meals and being overweight and obese, and to understand the relationship between maternal feeding style and the frequency of evening family meals. In this study of recent immigrant mother-child dyads, we found, similar to other studies, that having a high frequency of evening family meals is protective against child overweight and obesity. We also found that having a low demandingness feeding style was associated with a lower frequency of evening family meals. Targeting family meals as an intervention tool may help when designing obesity related interventions for predominately immigrant populations.

To our knowledge, this is the first report of family meal frequency being protective against child overweight and obesity among recent immigrants. These findings are similar to what has been found in predominately Caucasian, and some Hispanic and African American, populations [18,22,45]. Our findings support this relationship, which has also been shown in a diverse, nationally representative sample from the U.S. of preschool aged children, as well as in older children and adolescents [18-21]. Although there is strong evidence that family meals and overweight and obesity in children are negatively associated, there have been fewer studies exploring the possible contributing factors that may explain why some families have a higher frequency of family meals. One study using data from Project Eating Among Teens, found that a maternal high responsiveness/high demandingness parenting style was associated with more frequent family meals [25]. We found similar results in that a greater percentage of mothers with a high responsiveness/high demandingness feeding style reported frequent eating of evening family meals. We also found, in multivariate models, that mothers who had a low demandingness style, had a lower frequency of evening family meals. Although this has not been reported in the literature previously, it follows the reasoning that a mother who has a less demanding style of feeding may provide less structure and rules around family eating, and hence the family meal. Future studies should explore a mediation analysis between feeding styles, meal frequency and child weight status.

In our sample, it is likely that new immigrant mothers have other life stressors, such as employment, that compete with family meals, and this in turn influences the feeding environment [46]. In addition, given the high level of stress associated with the acculturative process, combined with a lack of social support, mothers may find it hard to engage in household routines such as family meals [47]. Given that parent–child relationships are bidirectional, it is also possible that children’s wishes may influence the mothers. Children of recent immigrant mothers may be more motivated to engage in certain “American” eating habits (e.g., eating in front of the TV, no scheduled meal time), which were unusual in their home country [48], and may pressure their mothers accordingly. Future work is needed to further understand how certain parental factors may positively and negatively influence the occurrence and frequency of family meals, and understand how parental and child factors interact to influence health behavior.

It is important to note that of those mothers who were employed, 75% reported having one or more jobs. Having a higher number of jobs was associated with a lower frequency of family meals. Conversely, mothers who reported either being unemployed or a student/homemaker reported a higher frequency of family meals. Future research is needed to help understand how much time is spent in meal preparation; how that and planning influences the occurrence of family meals; and how interventions may incorporate this knowledge.

Our findings should be interpreted within the context of the study limitations. Although our sample represents a diverse group of mother–child dyads, generalizability may still be limited given the focus on families from Brazil, Haiti and Latin America (predominately Central and South America). As a cross-sectional study, one can neither discern the direction of influence, nor look at change over time. For example, we do not know if family meal frequency was different before moving to the U.S., whether maternal feeding style was different in the home country, and whether maternal feeding style changed since moving to the U.S. Further, mothers are not parenting in isolation, but in response to many factors including child traits [49]. Thus, the parent–child relationship is bi-directional and additional studies that also include measures of specific child behaviors (e.g., food intake) are needed to understand causal pathways between parenting behaviors (especially from multiple caregivers) and child weight status. Also, although our measures captured some aspects of acculturation, we did not gather specific information about how the acculturative process may influence the dynamics of family feeding or other socio-cultural norms. Future studies should explore this process.

In conclusion, we found that evening family meals are protective against child overweight and obesity in this sample of immigrant families. This suggests that providing more structure and routine around mealtime may help promote healthy family practices.

## Competing interests

No competing financial interests exist.

## Authors’ contributions

All the authors contributed to the various stages of this study. AT contributed to the study design, performed all of the statistical analysis, and drafted the manuscript. EH contributed to the study design and helped draft and revise the manuscript. AP, AM, DG, and SH participated in the design of the study and revised manuscript. SS, RB, EKV, CLK and KT collected data and revised manuscript. CDE conceived of the initial idea of the study, contributed to design of the study, revised the manuscript and contributed especially to the intellectual content. All the authors read and commented on the drafts and approved of the final version for submission.
